# Projected climate change threatens significant range contraction of *Cochemiea halei* (Cactaceae), an island endemic, serpentine‐adapted plant species at risk of extinction

**DOI:** 10.1002/ece3.6914

**Published:** 2020-10-29

**Authors:** Peter B. Breslin, Martin F. Wojciechowski, Fabio Albuquerque

**Affiliations:** ^1^ School of Life Sciences Arizona State University Tempe AZ USA; ^2^ Science and Mathematics Faculty Arizona State University Mesa AZ USA

**Keywords:** biodiversity, biogeography, Cactaceae, climate change, endangered species, island endemism, range shifts, serpentine adaptation, species distribution modeling

## Abstract

**Aim:**

Threats faced by narrowly distributed endemic plant species in the face of the Earth's sixth mass extinction and climate change exposure are especially severe for taxa on islands. We investigated the current and projected distribution and range changes of *Cochemiea halei*, an endemic island cactus. This taxon is of conservation concern, currently listed as vulnerable on the International Union for the Conservation of Nature Red List and as a species of special concern under Mexican federal law. The goals of this study are to (a) identify the correlations between climate variables and current suitable habitat for *C. halei*; (b) determine whether the species is a serpentine endemic or has a facultative relationship with ultramafic soils; and (c) predict range changes of the species based on climate change scenarios.

**Location:**

The island archipelago in Bahía Magdalena on the Pacific coast, Baja California Sur, Mexico.

**Methods:**

We used temperature and precipitation variables at 30‐arc second resolution and soil type, employing multiple species distribution modeling methods, to identify important climate and soil conditions driving current habitat suitability. The best model of current suitability is used to predict possible effects of four climate change scenarios based on best‐case to worst‐case representative concentration pathways, with projected climate data from two general circulation models, over two time periods.

**Main conclusions:**

The occurrence of the species is found to be strongly correlated with ultramafic soils. The most important climate predictor for habitat suitability is annual temperature range. The species is predicted to undergo range contractions from 21% to 53%, depending on the severity and duration of exposure to climate change. The broader implications for a wide range of narrowly adapted, threatened, and endemic plant species indicate an urgent need for threat assessment based on habitat suitability and climate change modeling.

## Graphical abstract

The Cactaceae are the fifth most endangered plant or animal family on Earth, and many of those endangered species are narrowly distributed endemics. We investigated the impacts of climate change exposure on the distribution of a vulnerable island endemic cactus in Baja California, using species distribution models. We found that this species is likely to undergo a significant range contraction, which puts the species at risk of extinction over the next 50 years.

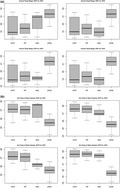

## 1 INTRODUCTION

Cactaceae are the 5th most endangered plant or animal family to be globally assessed to date by the International Union for the Conservation of Nature (Goettsch et al., 2015). The primary known threats to populations of cactus species are poaching of wild populations for the horticultural trade, small‐scale farming and ranching, mining operations, and the effects of climate change (Anderson et al., 1994; Bárcenas‐Luna, 2003; Godínez‐Álvarez et al., 2003; Goettsch et al., 2015; Hernández & Godínez‐Álvarez, 1994; Martorell & Peters, 2005; Oldfield, 1997; Téllez‐Valdés & Dávila‐Aranda, 2003; Ureta & Marti, 2012). Aside from these threats, increased risk of extinction in Cactaceae due to climate change exposure is not well understood (Goettsch et al., 2015). This study aims to help fill this gap, using predictive modeling to anticipate the extinction risk due to current conditions and climate change impacts, faced by an island endemic, threatened cactus.

Temperature and precipitation have been shown to be strong correlates for the distribution of plant species (e.g., Elith & Franklin, 2013; Guisan & Thuiller, 2005; Guisan & Zimmermann, 2000; Hawkins et al., 2003). Specifically relevant to this study, the biseasonal winter/summer precipitation cycles of the Sonoran Desert region, as well as longer precipitation cycles caused by shifts in the California Current, have been shown to drive plant and cactus distributions (Anderson, 2001). Cacti are often narrowly adapted to specific thermal niches, as well as highly sensitive to seasonal precipitation patterns (Gibson & Nobel, 1986). Also relevant to this study, since the study species is an island endemic, islands often have both thermal and precipitation differences from their nearest peninsular or continental landmasses (Humphreys et al., 2019; Kreft, et al., 2008). These factors have been shown to contribute to island endemism and increased risk to island biodiversity, not only for cactus species but also for species in general (Humphreys et al., 2019; Kreft et al., 2008). Islands harbor a significant amount of plant biodiversity (e.g., Kier et al., 2009; Kreft et al., 2008). Yet, island ecosystems also host endemic plant species subject to increased risk of extinction compared to the background rate (Humphreys et al., 2019).

Ultramafic soils (e.g., ophiolite, amphibolites, serpentine, and gabbros) have been shown to drive plant endemism and are often related to the distribution of *C. halei* Walton (Botha & Slomka, 2017; Kazakou et al., 2008; Kruckeberg, 1951). These soils contain high proportions of heavy metals and low quantities of plant nutrients and are toxic to most plant species. Consequently, species adapted to these soils have a competitive edge and can colonize areas that other plants cannot (Anacker et al., 2011; Brady et al., 2005; Harrison et al., 2006). To date, no studies of habitat suitability of cacti associated with ultramafic soils have been done, and the importance of this substrate to the distribution of *C. halei* is unknown.

Climate change is likely to affect the future distribution of many plant species due to shifts in temperature and precipitation (Bakkenes et al., 2002; Kelly & Goulden, 2008; Urban, 2015; Walther et al., 2002; Warren et al., 2018). In particular, climate projections under all representative concentration pathways (RCPs) of atmospheric “greenhouse gasses” and particulates show increased mean temperatures ranging from 1.5°C to 4.5°C globally and increased aridification of existing deserts due to larger areas subject to lower amounts of annual rainfall (Collins et al., 2013). Specifically concerning Cactaceae, prior to 2019, there were very few studies of habitat suitability and the potential effects of climate change on habitat suitability or distribution (Albuquerque et al., 2018; Butler et al., 2012; Martorell & Peters, 2005; Téllez‐Valdés & Dávila‐Aranda, 2003). Although cacti are adapted to arid conditions, prior studies have shown that they are nevertheless vulnerable to projected changes in both temperature and precipitation under climate change scenarios (Albuquerque et al., 2018; Butler et al., 2012; Martorell & Peters, 2005; Téllez‐Valdés & Dávila‐Aranda, 2003). The effect of climate change on the future suitable habitat of *C. halei* is unknown.

Our investigations include identifying the environmental variables that determine the habitat suitability of *C. halei*. Abiotic correlates for the distribution of rare, narrowly restricted endemic species can provide valuable insight into suitable habitat, possible threats to the persistence of populations, and the potential effects of future climate change (Benito et al., 2009; Franklin, 2010a, 2010b; Hawkins et al., 2003; Hijmans & Graham, 2006).

Our specific goals are to investigate the environmental correlates to the distribution of *C. halei*: (a) whether populations of *C. halei* are more likely to occur on ultramafic soil; (b) whether the species is expected to colonize the peninsula, or whether it is more likely to remain isolated on the islands; (c) and the effect of varying levels of climate change on the future range and as a contributor to the risk of local and global extinction of *C. halei* over the next 30–50 years. This study will help provide background for urgently needed future analyses of the specific risks faced by narrowly distributed, endemic, and endangered cacti and other island endemic plant species.

### 1.1 The study site

**Figure 1:**
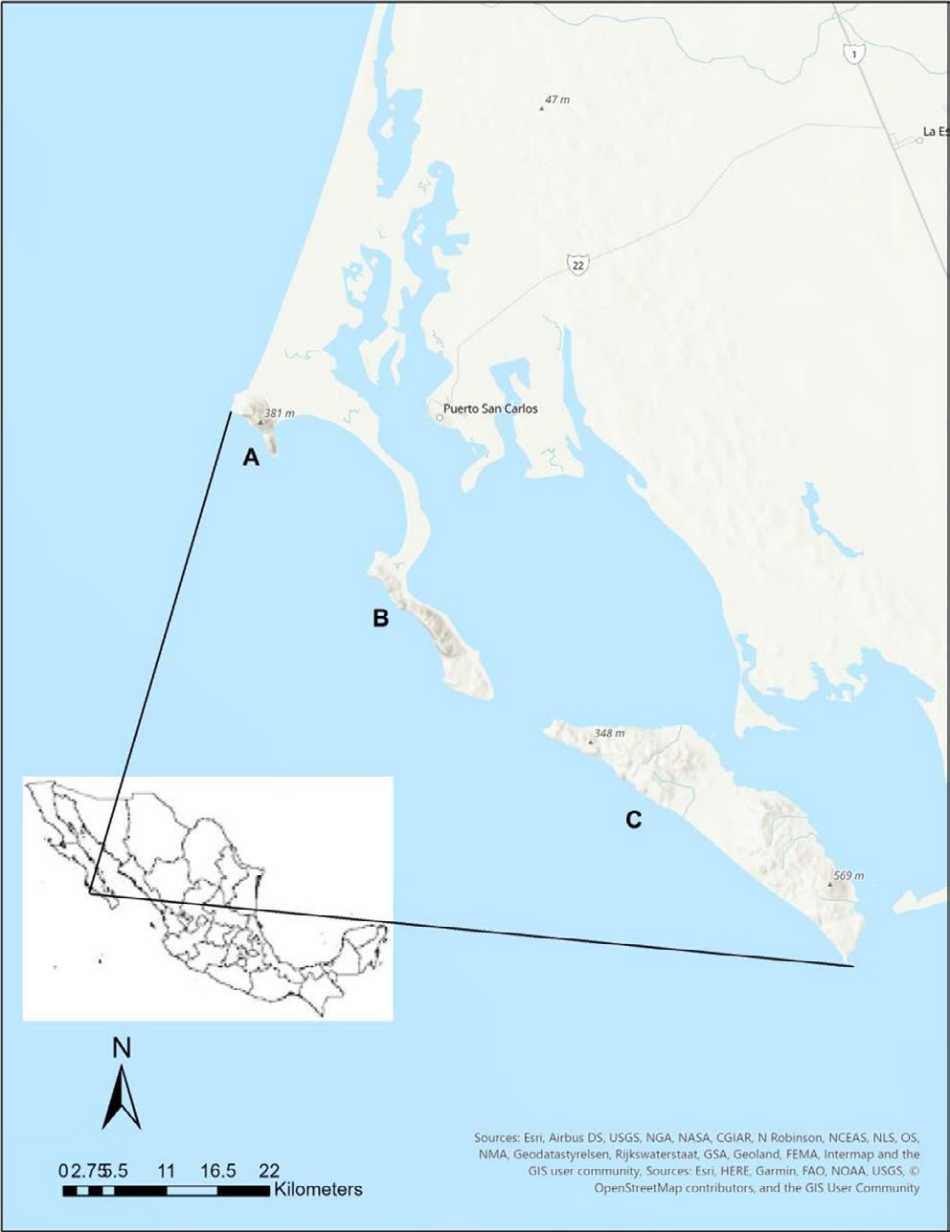
The Bahía Magdalena region and islands, the known area of distribution of *Cochemiea halei*. The islands and named land masses in Bahía Magdalena: (a) Cabo San Lazaro, the most northwesterly land mass of the islands. (b) The main land mass of Isla Magdalena. (c) Isla Margarita. Map created using ArcGIS® software by Esri. ArcGIS^®^ and ArcMap™ are the intellectual property of Esri and are used herein under license. Copyright^©^ Esri. All rights reserved

Bahía Magdalena is an ecologically significant embayment along the Pacific coast of the southern Baja California Peninsula (Bizzarro, 2008). In contrast to the adjacent coastal plains, the island archipelago in Bahía Magdalena is part of the North American Cordillera and has mountainous, rocky terrain as a result (Blake et al., 1984; Rangin, 1978; Sedlock, 1993) (Figure 1).

The islands range in elevation from sea level along the bay coastline to nearly 1,000 m (Blake et al., 1984). Several topographical variations on the islands create heterogeneous terrain, including flats, dunes, gravel coastlines, and highly eroded arroyos. Westerly cliffs drop to the Pacific, at angles as steep as 90^○^. These cliffs are predominantly exposed to ultramafic rock and gravel (Blake et al., 1984; Rangin, 1978).

The main soil mineralogical composition is serpentine rock and its eroded derivatives or nonultramafic basalt and sand (Blake et al., 1984; Rangin, 1978; Sedlock, 1993). The mountainous ridges consist of ultramafic, oceanic crustal rock formed through tectonic plate collisions estimated to have occurred from the Late Jurassic to the Late Cretaceous periods (Sedlock, 1993; Zaitsev et al., 2007).

Climate data from WorldClim v. 2.0 (Fick & Hijmans, 2017) shows biseasonal summer and winter precipitation, with autumn and spring being the dry months of the year. The moderating effects of the California Current System create narrower diurnal and annual temperature ranges, increased precipitation, and cooler seasonal averages for the islands than for the adjacent peninsula (Bakun, 1990; Bizzarro, 2008; Hickey, 1979; Robinson et al., 2007). From 2010 to 2018, 13 tropical storms or hurricanes occurred in the study region, with the majority occurring in the hottest month, September (National Hurricane Center; Hurricane Research Division; Central Pacific Hurricane Center).

The vegetation of the Bahía Magdalena region features 18 endemic angiosperm taxa; the endemic cacti represent 33% of the plant endemism in the area (León de la Luz et al., 2015). The area is recognized as one of nine regions of high plant endemism in Baja California (Reimann & Ezcurra 2005). The primary vegetative regime is fog crassicaulescent and sarcocaulescent scrub, that is, a combination of leaf and stem succulents, such as the endemic *Agave margaritae* Brandegee and scrub vegetation, generally <8 m high (Rebman & Roberts, 2012, León de la Luz et al.).

**Figure 2:**
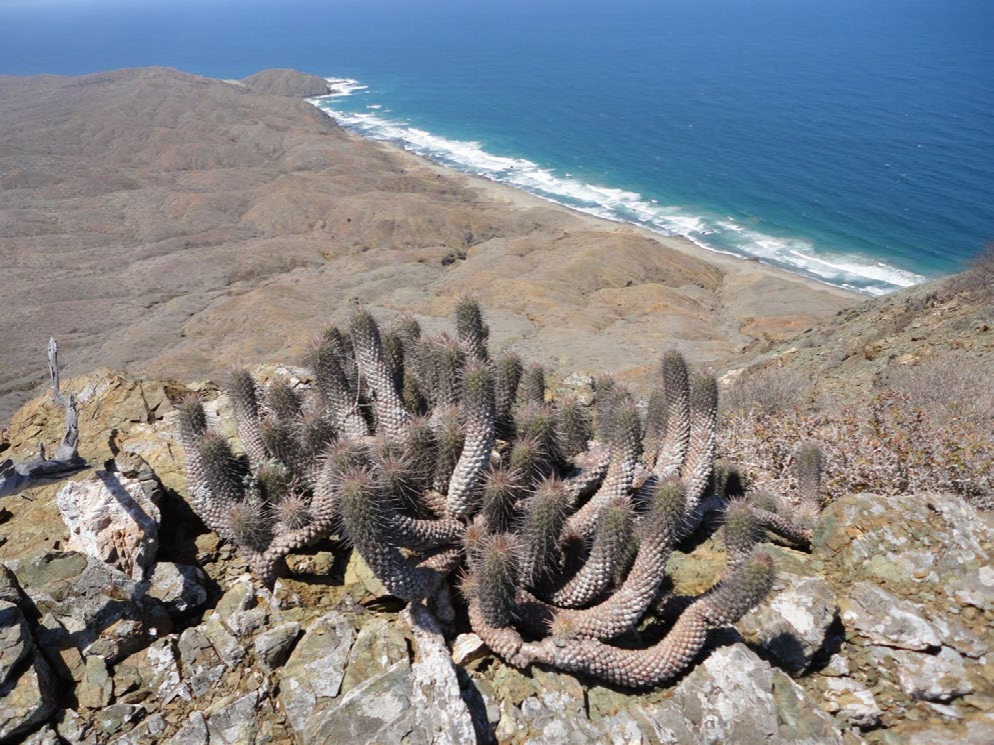
*Cochemiea halei* in habitat on Isla Magdalena, growing in pure ultramafic rock

*Cochemiea halei*, the study species, is a mat‐forming stem succulent with straight spines and presumably hummingbird‐pollinated flowers (Craig, 1945; Pilbeam, 1999) (Figure 2). *Cochemiea halei* is of conservation concern, assessed as vulnerable by the IUCN, and protected by Mexican law. The factors leading to its classification as vulnerable are the narrow geographic range, the low overall population size, and evidence of declining populations (IUCN). No formal studies of its population viability or quantified risk of extinction have been conducted previously, however.

## 2 METHODS

### 2.1 Survey methods

Occurrence data were gathered over four years with eight surveys, in both winter and spring. Random points within the smallest convex polygon around Isla Magdalena and Isla Santa Margarita were generated using ArcMap (v. 10.6.1, ESRI, Inc.). The random points were used as centers of survey transects (Bonham, 2013; Elzinga et al., 1998). In addition to occurrence data, we recorded soil type data and other topographical information. The latitude and longitude of occurrences were marked using a handheld GPS device (eTrex 30X, Garmin Ltd.). A different surveying technique was employed on Cabo San Lazaro due to the much smaller area of that landmass. At that site, a belt transect method was used, with individuals counted within a 300 m radius along a 3 km segment that covered the entire habitat of *C. halei* (Bonham). The only known peninsular population, with six individuals (Gorelick, 2007), was included in all analyses.

Presence points were spatially thinned to a minimum separation of 1 km, the resolution of our climate data, to eliminate spatial autocorrelation effects during modeling (Stolar & Neilsen, 2015; Tessarolo et al., 2014). Point pattern analysis was performed using Ripley's *K* statistic to measure the degree of spatial correlation of presence/absence records (Baddely, 2008).

### 2.2 Environmental variables

To investigate correlations between the distribution of *C. halei* and its environment, we chose 19 temperature and precipitation variables from WorldClim v. 2.0, averages from 1970 to 2000, at 30‐arc sec resolution (Fick & Hijmans, 2017). Soil type was determined during field surveys using a Munsell soil identification color scale (Munsell Color, Grand Rapids, MI), categorizing soils into ultramafic (2.5Y hue with various color values and chroma) versus either “nonserpentine” (approximately 7.5YR to 10YR) or sand (Roberts, 1980). A dense sampling of occurrences of *C. halei* with soil type data was performed to reduce error when interpolating for missing values (Carl & Kühn, 2007; Dormann et al., 2013; Dormann & McPherson, 2007). The soil type data from the field was mapped onto zones of ultramafic versus nonultramafic substrate, as indicated in the geological map of Isla Magdalena and Isla Margarita by Rangin (1978). The soil type raster was generated using inverse distance weighted interpolation (Gonçalves, 2006; Grunwald, 2009) and improved using root‐mean‐squared error and fivefold cross‐validation (Gonçalves, 2002).

Four RCPs were used in climate change projections: 2.6, representing the best‐case future concentration of carbon in the atmosphere, through intermediate levels 4.5 and 6.0, to the worst‐case scenario of 8.5, as outlined in the Intergovernmental Panel on Climate Change's Fifth Assessment Report (IPCC, 2013; Liddicoat et al., 2013). The climate data itself were derived from two general circulation models (GCMs). The GCMs used were the Hadley Center Global Environmental Model version 2‐ES (HadGEM2‐ES) and the Community Climate System Model v. 4 (CCSM4), both of which are frequently used in studies of climate change effects on habitat suitability (e.g., Albuquerque et al., 2018; Bellouin et al., 2011; Leclère et al., 2014; McQuillan & Rice, 2015). The HadGEM2‐ES model scenarios include projections of changes in ocean temperature and sea ice, and are especially recommended for use in predicting changes in coastal habitat (Caesar et al., 2013; Collins et al., 2008).

### 2.3 Variable preparation

The reduction of multicollinearity for all variables was performed by constructing a correlation matrix and performing hierarchical cluster analysis, which groups variables according to their mutually related correlations (Albuquerque et al., 2018; Benito et al., 2013; Sarstedt & Mooi, 2014). A cutoff of 0.5 Pearson's correlation index was used; all variables correlated higher than 0.5 were discarded (Albuquerque et al., 2018). The biserial correlation analysis, with variables correlated to presence/absence data for *C. halei*, was performed for all variables below 0.5 (Albuquerque et al., 2018; Kraemer, 2006; Stolar & Nielsen, 2015). From each cluster of correlated variables, as derived from the hierarchical cluster analysis, the variable with the highest correlation to the distribution of *C. halei* was chosen for use in modeling.

### 2.4 Modeling methods

Three methods were used for building models: boosted regression trees (BRTs), generalized linear models (GLMs) of the binomial family, and maximum entropy (Maxent) (Elith et al., 2008; Franklin, 2010a, 2010b; Hijmans & Graham, 2006). Models used field survey presence and 200 randomly generated pseudoabsence background points (Elith & Franklin, 2013; Elith et al., 2010; Franklin, 1995, 2010; Guillera‐Arroita et al., 2015; Phillips & Elith, 2013). In each run for all model methods, the presence/pseudoabsence data used were post‐thinning, with no more than one occurrence or pseudoabsence per 1‐km grid square.

Boosted regression trees is an iterative machine learning optimization method, in which the deviance residuals from a prior decision tree are used as the data for the next step (called “boosting”); the decision tree building process continues until residual deviance is no longer decreased by iterations (De'ath, 2007; Franklin, 2010a, 2010b). Decision trees, the underlying algorithm of BRT, also known as classification and regression trees, perform well with both continuous and categorical variables, and, unlike with GLM, for example, they are robust to a lack of independence among predictors (Albuquerque et al., 2018; De'ath, 2007; Elith & Leathwick, 2009, 2017). GLM is a well‐known regression method that uses maximum likelihood as the measure of the contribution of a variable to a prediction of the “state” of a dependent variable, in this case, the binary outcome of presence/absence (Guisan et al., 2002; Nelder & Wedderburn, 1972). Maximum entropy (Maxent) is a machine learning method that employs multinomial logistic regression to estimate the probability of the distribution of a species according to the “maximum entropy” of the distribution, that is, the most uniform distribution of a species possible given the limits imposed by the predictor variables (Elith et al., 2011; Phillips et al., 2017; Phillips et al., 2006).

### 2.5 Model evaluation

For BRT models, evaluation of model performance included measures of residual deviance, k‐fold cross‐validation, and the area under the receiver operator characteristic curve (AUC) (De'ath, 2007; Elith & Leathwick, 2009). GLM performance was evaluated with AUC and adjusted *D* ^2^ (a measure of the difference between null deviance and model deviance adjusted for degrees of freedom) and Akaike's information criterion (Franklin, 2010a, 2010b; Guisan et al., 2002). Maxent models were evaluated by comparative training and test AUC, and omission on test and training samples against random prediction (specificity–sensitivity curves) (Elith & Leathwick, 2017; Hijmans, 2012).

Current habitat suitability predictions using soil type were compared to predictions with soil type removed. The predictions with soil type were subtracted from the predictions without soil type, to find the percent changes in suitable habitat when soil type was not used as a predictor. These percent changes were mapped over the study site and compared to the presence or absence of ultramafic soils.

### 2.6 Climate change modeling

Climate change scenarios were projected using the best predictive model for current habitat suitability, for the periods 2009–2049 and 2009–2069. The current predicted suitable habitat was subtracted from composite binary presence/absence maps using both GCMs. Range differences counted as “contractions” if a current presence was projected as a future absence, “refuge” if current presences remained presences, and “expansion” if the current unsuitable habitat was projected as suitable in the future (Albuquerque et al., 2018; Elith et al., 2010; Hatten et al., 2016).

## 3 RESULTS

### 3.1 Survey results

A total of 1,227 records were recorded in the field, with accompanying soil type. After separating occurrence points by a minimum distance of 1 km, and removing duplicate records, the occurrence/pseudoabsence data set used in modeling consisted of 44 presences and 207 pseudoabsences.

### 3.2 Variable selection

The variables that were below the 0.5 correlation threshold in the cluster analysis but most strongly correlated to the occurrence of *C. halei* in the biserial correlation analysis were annual temperature range, mean temperature of the warmest quarter, precipitation of the warmest quarter, and precipitation of the coldest quarter. These variables, along with soil type, were used in the species distribution models. Annual temperature range differs on the islands versus the peninsula, with island temperature ranges of approximately 20°C and the peninsular range 10° wider at 30°C. Mean temperature of the warmest quarter ranges from 24°C in the northwestern region of Isla Magdalena to approximately 30°C at the lowest elevation in the center of Isla Margarita and on the peninsula. Precipitation of the warmest quarter ranges from 20 to 40 mm on the islands, except for the highest elevations on Isla Margarita at 70 mm. The majority of peninsular precipitation is also at 70 mm. Precipitation of the coldest quarter is from 20 to 30 mm on the islands to slightly over 40 mm on the peninsula.

### 3.3 Modeling results

The three modeling methods of GLMs, BRTs, and maximum entropy all generated fair to excellent results, as measured by AUC values, with values ranging from 0.75 to 0.95. Boosted regression trees produced the most informative models, as well as models that projected the best to future climate change scenarios. Maxent models had low predictive power, probably a result of the small spatial scale of the study area, with the best performing models having an AUC of 0.75. Generalized linear models had the highest AUC values at ~0.95, but the models were highly overfitted, and as a result, projecting them to climate change scenarios was not informative.

**Figure 3:**
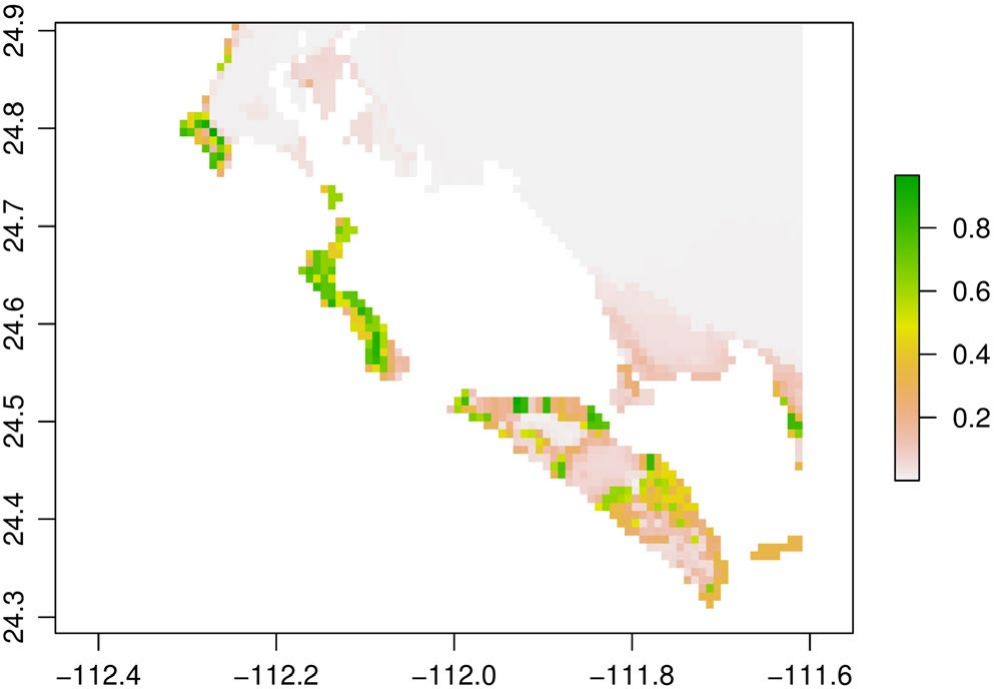
Predictions of suitable habitat for *Cochemiea halei*. The map shows predictions of habitat suitability, on a probability scale of zero (transparent) to 1 (dark green). The model predictions derive from a BRT method, using WorldClim V. 2.0 data, at 30‐arc sec resolution. 44 presences and 207 pseudoabsences were used. The following variables were used: annual temperature range, the mean temperature of the warmest quarter (July–September), precipitation of the warmest quarter (July–September), precipitation of the coldest quarter (December–February), and soil type. The model fitted 11,125 trees, with a 10‐fold cross‐validated AUC of 0.96. The parameters used for the boosted regression tree analysis were a tree complexity of 2, a learning rate of 0.0007, bag fraction of 0.7, and a step size of 25

As a result, the model results presented below are derived from the BRT analyses, which had good AUC values of 0.85–0.88, projected with lower error to future climate conditions, and provided informative estimates of the influence of predictors on habitat suitability and possible future range changes. The first model presented here is a model that included soil type, which was the best performing BRT model (Figure 3).

**Figure 4:**
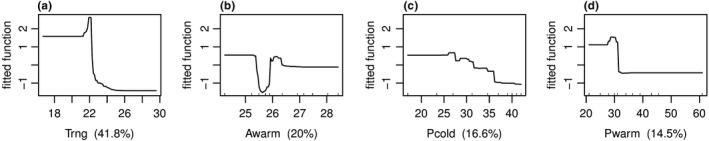
Partial response plots of climate variables used in species distribution modeling for *Cochemiea halei*. The plots show the marginal response of *C. halei* to each variable. The variables are (a) annual temperature range, “Trng,” (b) average temperature of the warmest quarter, “Awarm,” (c) precipitation of the warmest quarter, “Pwarm,” and (d) precipitation of the coldest quarter. The *y*‐axis for each plot is on a logit scale, showing the relative impact of values of the variable on the probability of occurrence. The *x*‐axis for the temperature variables, (a) and (b), is in degrees C. Precipitation variables, (c) and (d), are in mm. The *x*‐axis is marked with a decile rug plot

*Cochemiea halei* shows strong partial responses to the predictors used in modeling. Annual temperature range has the most significant impact on current habitat suitability, with a sudden drop in suitability under an annual temperature range greater than approximately 21.5°C. Partial response plots also specify the response of *C. halei* to other predictors. Mean temperature of the warmest quarter ranges from 24°C to 26°C, with an increased contribution to occurrence at 26°C, but then a sharp drop‐off, with temperatures above approximate 26.5°C negatively correlated to occurrence. Suitable habitat is positively correlated to precipitation of the warmest quarter below 30 mm, and negatively, above 30 mm. Precipitation of the coldest quarter shows approximately the same response of precipitation of the warmest quarter (Figure 4).

**Figure 5:**
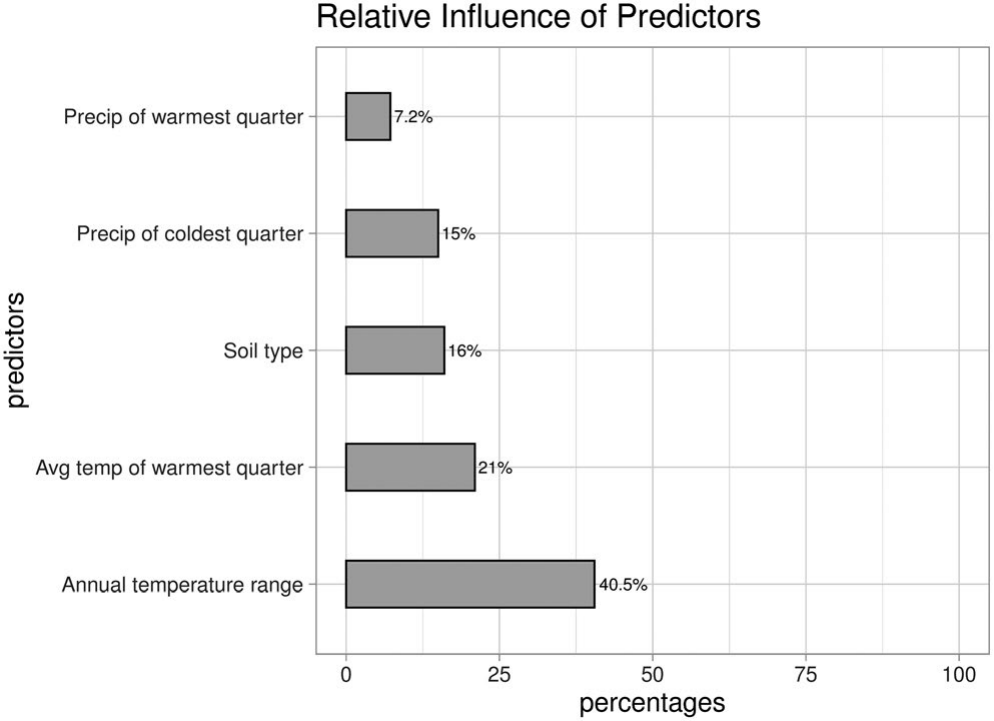
Percent contributions of climate predictors to model performance in estimating the current suitable habitat of *Cochemiea halei*. Percent influence: annual temperature range, 40.5%; average temperature of the warmest quarter, 21%; soil type, 16%; precipitation of the coldest quarter (December–February), 15%; precipitation of the warmest quarter (July–September), 7.2%

The percent relative contributions for each variable to the predictive ability of the model described above show that the most significant predictors are annual temperature range and average temperature of the warmest quarter, accounting for nearly 62% of model performance. (Figure 5).

**Figure 6:**
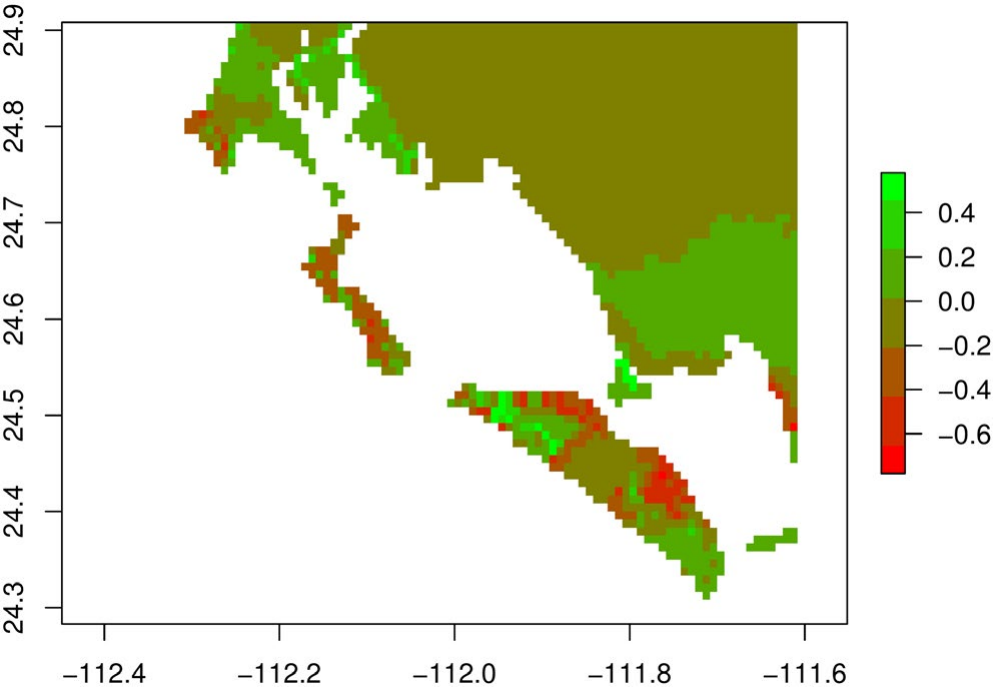
Percent changes in predicted current suitable habitat of *Cochemiea halei*, with soil type and without soil type. Values on the scale bar and their corresponding colors on the map show percent increase or percent decrease in predicted probability of occurrence when soil type is not used as a predictor. The majority of the map predicts an increase in the probability of occurrence (green colors), especially in areas where ultramafic soils are not predominant. Also, the model without soil type predicts less suitable habitat (red), in some areas that are predominantly ultramafic soils. The best model without soil type fitted 12,650 trees, with a 10‐fold cross‐validated AUC of 0.95. The parameters used for the boosted regression tree analysis were a tree complexity of 1, a learning rate of 0.0007, bag fraction of 0.7, and a step size of 15

To gauge the impact of soil type on the habitat suitability of *C. halei*, a model was created with the same predictor variables as the above model, but without soil type. The map of percent changes in suitable habitat when soil type is removed shows increased probabilities of occurrence on nonultramafic soils, as well as some decreases in the probability of occurrence on ultramafic soils. Higher suitability is also predicted within the islands themselves overall, with a higher probability in general of suitable habitat. Some pure sand features, such as the sand that connects Isla Magdalena with Cabo San Lazaro, where *C. halei* is not known to occur, are also predicted to have increased suitability when soil type is not used (Figure 6).

### 3.4 **The effects of climate change on the range of *C*** *.* ***halei***

**Figure 7:**
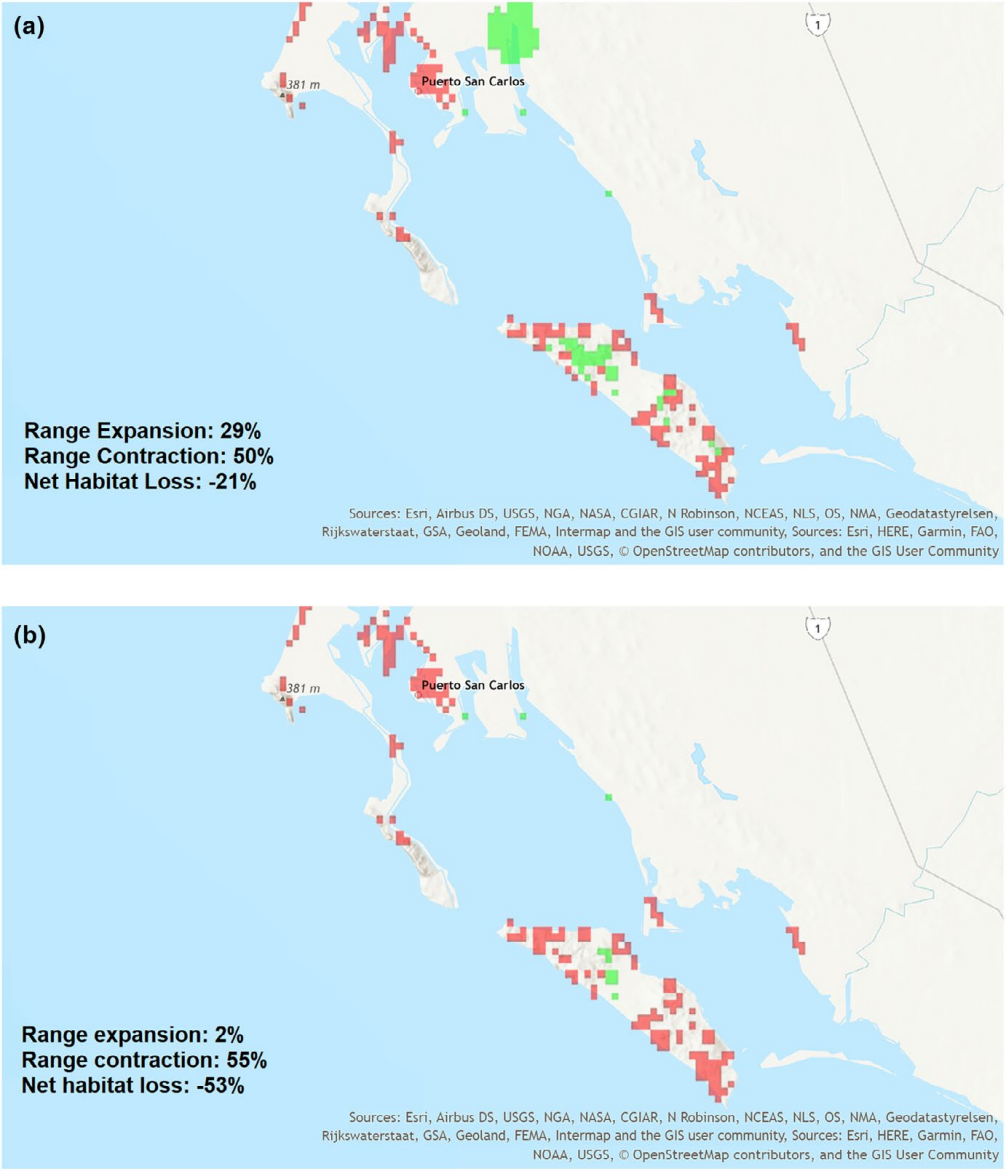
Predictions of range expansion and contraction for *Cochemiea halei*, to the year 2070, using two representative concentration pathways. Light green areas represent projected areas of range expansion, and red areas represent projected areas of range contraction. (a) predicted expansion and contraction for a representative concentration pathway (RCP) of 2.6, representing relatively low climate change exposure, and (b) predicted expansion and contraction for RCP 8.5, representing a high degree of climate change exposure. The model used for projection was the best performing BRT model with soil type. Projections were done using the mean values from the same climate variables used in habitat suitability modeling, using the Hadley Center Global Environmental Model version 2‐ES (HadGEM2‐ES) and the Community Climate System Model v. 4 (CCSM4).

For all projections, loss of between 21% and 53% of suitable habitat is predicted for the species. In the case of the lower RCPs, range contraction is partially offset by expansion into a previously unsuitable habitat. As the climate change scenarios increase in RCP, especially over the longer period to 2070, expansion is reduced significantly (Table 1). The range maps showing projected future areas of contraction and expansion for *C. halei* indicate the greatest potential loss of habitat is on Isla Margarita, with regions on that island accounting for 40%–65% of the total contraction (Figure 7).

**Table 1:** Percent expansion, contraction, and net habitat loss of *Cochemiea halei* under four representative concentration pathway (RCP) climate change scenarios and two time periods

Time period	RCP	Expansion (%)	Contraction (%)	Net habitat loss (%)
2050	2.6	19	−46	−27
	4.5	10	−48	−38
	6.0	14	−47	−33
	8.5	25	−52	−27
2070	2.6	29	−50	−21
	4.5	5	−50	−45
	6.0	6	−50	−44
	8.5	2	−55	−53

**Figure 8:**
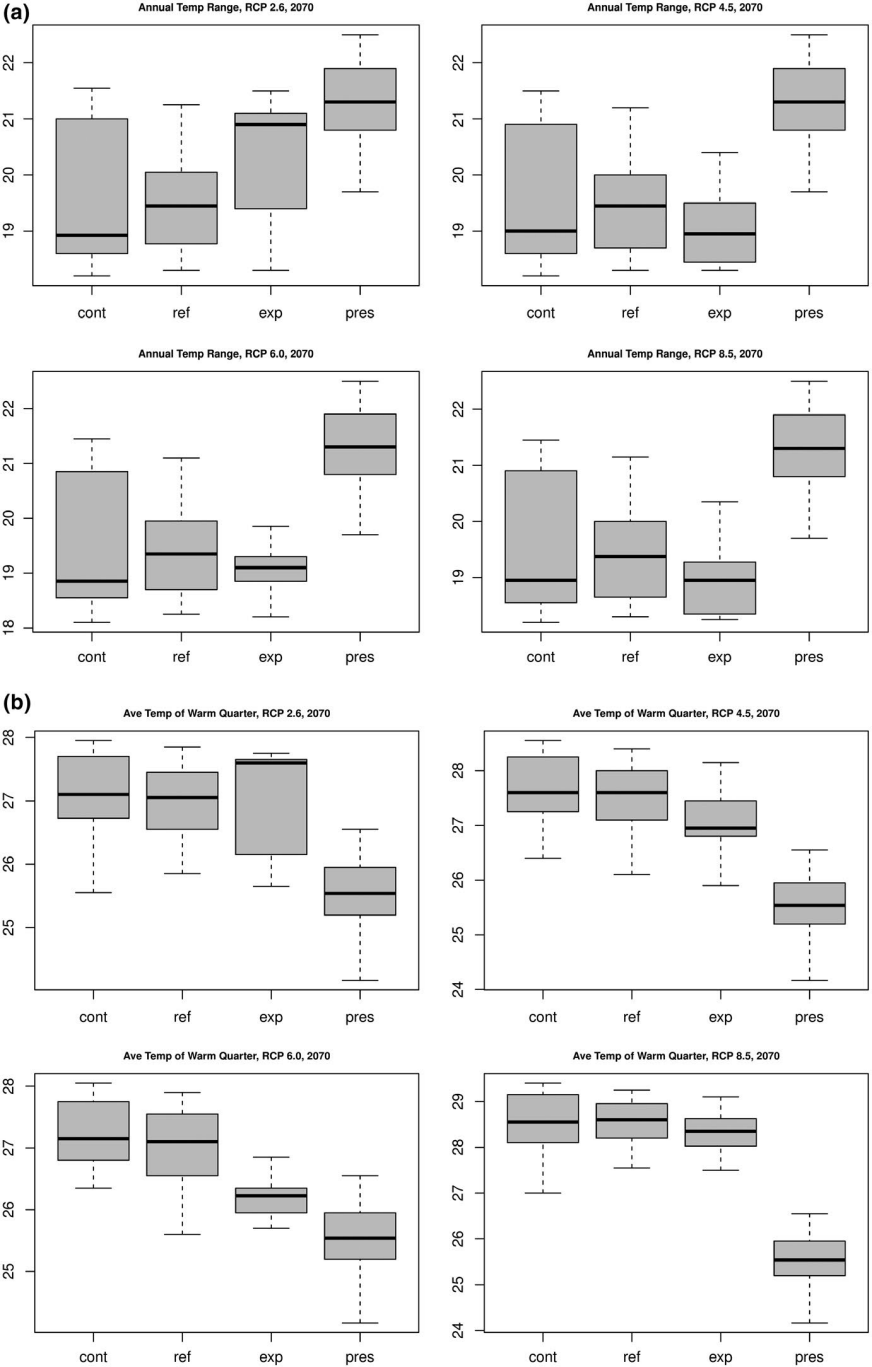
Influence of the two strongest climate predictors on habitat contraction, refuge, and expansion, for *Cochemiea halei*, for four representative concentration pathway scenarios, projected to 2070. Each box plot shows the range, 1st quartile, median, and 3rd quartile of annual temperature ranges and average temperature of the warmest quarter for each type of predicted future habitat prediction: contraction, refuge, expansion, and the present habitat. The *y*‐axes are degrees C. All data are from the projections to 2070.

Box plots of the effect of the two most important predictors, annual temperature range and mean temperature of the warmest quarter, on future habitat suitability indicate significant changes for all climate change projections to 2070. The greater variability of the annual temperature range for range contraction areas is consistent with the species having a more suitable habitat within a narrower temperature range. The significantly higher mean temperatures of the warmest quarter for all projected future areas also contribute to habitat loss. Predicted areas of expansion also feature higher temperatures, which is a result of temperatures across the study site increasing due to climate change (Figure 8).

## 4 DISCUSSION

We investigated the effects of environmental and soil variables on the distribution of *C. halei*, as well as the possible impacts of climate change, using species distribution models. Our results show the current distribution of the species and potential threats to the persistence of the species under climate change as a result of range contraction. *C. halei* has a fragmented predicted current suitable habitat on the islands, does not have suitable habitat on the peninsula, and is projected to lose as much as 53% of its current suitable habitat under climate change exposure.

The island endemism of *C. halei* is strongly correlated with both soil and climate effects. The island archipelago in Bahía Magdalena, the primary suitable habitat for *C. halei*, has different soil and climate from the nearby peninsula. These contrasting conditions, where conditions are significantly different even a short distance inland, are also found in other island habitats near coastal areas along the Pacific Ocean, (Bizzarro, 2008; Ratay et al., 2014; Reimann & Ezcurra, 2005).

*Cochemiea halei* occupies a narrow range of temperature and precipitation correlates, as seen in the partial response plots to the predictor variables. The moderating temperature effects of the California Current System (Bakun, 1990; Hickey, 1979; Huyer, 1983; Robinson et al., 2007) are important to habitat suitability for the species. These climate effects do not extend to the nearby peninsula to the degree that they are represented on the islands. The two most influential climate variables in the best model were annual temperature range and average temperature of the warmest quarter, accounting for approximately 73% of the model's predictive power. Both of these variables show significantly lower values on the islands than on the peninsula, patterns typical of coastal areas moderated by the upwelling of the California Current System, especially in summer (Bakun, 1990). The temperature range on the islands is approximately 10°C narrower than on the peninsula, and the mean temperature of the warmest quarter is 4°C cooler. The biseasonal precipitation patterns of the Sonoran Desert Region (Shreve & Wiggins, 1964; Burquez et al. 1999) are represented by the influence on the model performance of the precipitation of both the warmest and coldest quarters. However, there is less precipitation on the islands than inland, during both seasons, with the most significant differences occurring during the warmest quarter. These localized effects have been shown to drive endemism (Gogol‐Prokurat, 2011; Hijmans & Graham, 2006; Snyder et al., 2003; Humphreys et al., 2019). *C. halei* is an example of a species that has a localized, well‐defined climate response, with the highest probability of suitable habitat predicted to be within a relatively narrow band of thermal and precipitation parameters, characteristic of the islands where it is found.

In addition to strong climate influences on the current distribution of *C. halei*, soil type plays an important role. Narrowly restricted endemic plant species, including cacti, are strongly dependent on soil types for habitat suitability (Harrison et al., 2006; Kruckberg & Rabinowitz, 1985; Kruckeberg, 1951). Additionally, several studies of plant distributions have determined the importance of ultramafic soils in particular as a driver of plant endemism (e.g., Kruckberg, Botha & Slomka, 2017; Kazakou et al., 2008). While *C. halei* does not appear to be an obligate endemic to ultramafic soils, the species is more likely to occur on those soil types, with 60% of occurrences on ultramafic soil. This is similar to other species in the Cactaceae that occur on ultramafic soils, in particular on the islands of Cuba and Puerto Rico (Reyes‐Fornet et al., 2019). Obligate and facultative adaptations to ultramafic soils have been shown to provide a competitive advantage (Anacker et al., 2011; Brady et al., 2005; Harrison et al., 2006; Pollard et al., 2014). Although soil type is not as influential on model performance as temperature, the model with soil type had stronger predictive performance and indicated a more fragmented, lower habitat suitability for areas in our surveys where observed population density was low. This suggests that ultramafic soils are an important constraint on the distribution of the species.

*Cochemiea halei's* observed establishment on virtually unweathered ultramafic rock and exposed gravel, in addition to lower precipitation on the islands than on the peninsula, suggests that the species is adapted to evaporation of soil moisture and drier conditions, a common characteristic of cacti distributed in rocky environments (Gibson & Nobel, 1986). In summary, *C. halei* favors cooler, drier habitat, on ultramafic rock and soil, with a moderated annual temperature range, a suite of abiotic predictors that are the hallmarks of the island habitat, in contrast to the nearby peninsula.

Our models show that *C. halei* is not likely to disperse to the peninsula, except for small foothold regions along the peninsular shore. A molecular phylogeny and biogeography of the subclade and clade in which this species is nested (P.B. Breslin, M. F. Wojciechowski, & L.C. Majure, in prep.), indicate that geographical features, even on a large scale, such as the entire Gulf of California or the breadth of the peninsula at its widest points, are not a barrier to dispersal for this clade. The most likely dispersers of *C. halei* are frugivorous birds (Jon Rebman, pers. comm., 2019), and the maximum distance from the islands to the peninsula, at approximately 20 km, is no barrier to seed dispersal for bird species (Bregman, 1988; Fleming & Sosa, 1994; Rojas‐Arichiga & Vazquez‐Yanes, 1999). Further evidence that the island climate and soil limit the distribution of *C. halei*, and not dispersal, is found in the total absence of the species in the low lying trough on Isla Margarita, which has an absence of ultramafic soils, and vegetation identical to the Magdalena Plains on the peninsula (León de la Luz et al., 2015). Despite *C. halei's* absence in this trough, it is common at a higher elevation, rockier habitat fewer than 3 km to the northwest and southeast (Breslin, personal obsv.). Yet more evidence that climate and soil drive the isolation of *C. halei* on the islands rather than dispersal, the only known peninsular population of *C. halei* consists of approximately six individuals limited to a patch of sand measuring 150 m^2^. At that site, there is no sign of dispersal in the surrounding area, despite the plants being large, seed‐bearing, having several square km of available habitat, and having been established at this single site for at least 50 years (Gorelick, 2007). This characterizes *C. halei* as most likely a “stranded” endemic, making its persistence more vulnerable to changes in climate (Cowie & Holland, 2006; Crawford & Stuessy, 1997; Stuessy et al., 2014).

The suitable habitat for *C. halei* is a patchwork of sites even within its narrow range on the islands. Major geographical distinctions within the islands that are illustrated on the prediction map from the model with the best predictive ability (Figure 4) include two distinct regions on Isla Margarita, zones of less suitable habitat on Isla Magdalena, and a narrow zone of suitability at Cabo San Lazaro, with few areas of on‐the‐ground connectivity between suitable habitats. A population viability analysis of *C. halei* using deterministic and stochastic matrix population models with habitat‐specific sampling (Breslin, unpublished research) found that the species is most vulnerable to extinction over the next 100 years in low elevation, hotter habitat. This finding is supported by the results here, which show that most of the loss of suitable habitat for the species occurs at lower elevations. It may be that the extremes of climate that drive suitable habitat for *C. halei* are not captured within the low elevation areas of the islands, and future experimental studies would clarify that question. However, endemic plant species, such as *C. halei*, often occur in fragmented habitat with geographical barriers and low connectivity between sites (Kotliar & Wiens, 1990; Rabinowitz, 1981). As a narrowly restricted endemic, essentially stranded on the islands, the species is at increased risk for stochastic environmental, demographic, and genetic setbacks (Ellstrand & Ellam, 1993; Lande, 1993; Matthies et al., 2004; Melbourne & Hastings, 2008; Menges, 1992; Mubayi et al., 2019). Even without the impacts of climate change exposure, the species appears to be at elevated risk for local extinction events, population bottlenecks, and increased fragmentation.

### 4.1 Climate change

Future climate change scenarios indicate a contraction of *C. halei’s* range of 21% to 53%, depending on the severity of climate change and the length of time the species is exposed to climate change effects (Table 1). The range contraction reduces suitable habitat on the islands, and the species is unlikely to expand to the peninsula within the climate change conditions and periods projected here. The unique adaptations of narrowly distributed endemic plant species such as *C. halei* also make them vulnerable to changing climate conditions, as those adaptations are often in response to significantly different local climates or soil types that are unsuitable for related species (Damschen et al., 2012).

Specifically, each of the climate change scenarios projected in this study indicates a widening of the annual temperature range on the islands, which significantly decreases suitable habitat and reduces areas of expansion. The areas of predicted contraction under all scenarios are lower elevation, mostly bayside, leeward flats (Figures 7 and 8). The areas of expansion are mostly into the higher elevation ridges, especially on Isla Margarita. But the opportunity to expand into these higher elevation locales is greatly reduced as climate change becomes more severe or persists for a longer period.

Projections under all climate change scenarios are for a higher mean temperature of the warmest quarter, ranging from approximately 3°C to as high as 6°C. Precipitation of the warmest and coldest quarters is projected to decrease by 10 mm to 15 mm for regions of predicted range contraction. The wider thermal span and the warmer mean temperature from July–September, along with reduced precipitation, are combined factors that contribute to range contraction, driven by hotter, drier climate. Predicted range contractions are consistent with *C. halei's* narrow adaptation to a distinct island climate.

Caution in interpreting our results is necessary due to the small spatial scale of our study site compared to the 30‐arc sec resolution of the climate data we used. This resolution, providing climate surfaces with approximately 1‐km grid squares, is currently the highest resolution climate data available for our study site. Interpolation of climate data introduced unacceptable levels of error. Downscaling was not an option for our study site, due to a lack of weather stations or available long term climate data in the region. Nevertheless, 30‐arc sec climate data provided strong model performance and was sufficient to address the research questions in this study. This is probably primarily due to the distinct differences between the island climate and soil versus these variables on the peninsula, a result of the highly localized effects of the California Current System and the geological composition of the islands.

For the first time, temperature and precipitation correlates are identified that drive the fragmented, highly restricted distribution of an island endemic, vulnerable cactus. We used multiple modeling methods to determine the correlations between topographical and climate variables and the habitat suitability of *C. halei*, a little‐studied, island isolated cactus. Our results support the following conclusions: (a) Both the moderating effects of Pacific Coastal island climate and ultramafic soils unique to the islands strongly determine suitable habitat, which is fragmented, (b) the species is unlikely to disperse to the peninsula, (c) the species has a facultative but not obligate relationship with ultramafic soils, and (d) climate change in all scenarios is likely to contract the range of the species, as a result of greater variability in annual temperature range, higher mean temperatures in the summer, and reduced precipitation. Our findings indicate that this narrowly restricted endemic cactus is at increased risk of extinction, and populations should be carefully monitored over at least the next 50 years.

## CONFLICT OF INTEREST

None declared.

## AUTHOR CONTRIBUTION

**Peter B Breslin:** Conceptualization (lead); Data curation (lead); Formal analysis (equal); Funding acquisition (lead); Investigation (lead); Methodology (equal); Writing‐original draft (lead); Writing‐review & editing (equal). **Martin Wojciechowski:** Formal analysis (equal); Methodology (equal); Project administration (equal); Supervision (lead); Writing‐review & editing (equal). **Fábio Albuquerque:** Conceptualization (equal); Data curation (equal); Formal analysis (equal); Investigation (equal); Methodology (equal); Supervision (equal); Writing‐review & editing (equal).

## Data Availability

The raw data that support the findings of the study are available at Dryad, DOI https://doi.org/10.5061/dryad.t4b8gtj0d
